# Autoantibodies to angiotensin and endothelin receptors in systemic sclerosis induce cellular and systemic events associated with disease pathogenesis

**DOI:** 10.1186/ar4457

**Published:** 2014-01-28

**Authors:** Angela Kill, Christoph Tabeling, Reinmar Undeutsch, Anja A Kühl, Jeannine Günther, Mislav Radic, Mike O Becker, Harald Heidecke, Margitta Worm, Martin Witzenrath, Gerd-Rüdiger Burmester, Duska Dragun, Gabriela Riemekasten

**Affiliations:** 1 German Rheumatism Research Centre (DRFZ), A Leibniz Institute, Berlin, Germany; 2 Department of Rheumatology and Clinical Immunology, University Hospital Charité, Luisenstraße 13, Berlin 10117, Germany; 3 Department of Infectious Diseases and Pulmonary Medicine, University Hospital Charité, Berlin, Germany; 4 Department of Inner Medicine, University Hospital Charité, Berlin, Germany; 5 Department of Rheumatology and Clinical Immunology, University Hospital Split, Split, Croatia; 6 CellTrend GmbH, Luckenwalde, Germany; 7 Department of Dermatology, University Hospital Charité, Berlin, Germany; 8 Department of Nephrology and Intensive Care Medicine, University Hospital Charité, Berlin, Germany

## Abstract

**Introduction:**

Vasculopathy, inflammatory fibrosis and functional autoantibodies (Abs) are major manifestations of systemic sclerosis (SSc). Abs directed against the angiotensin II type 1 receptor (AT_1_R) and endothelin-1 type A receptor (ET_A_R) are associated with characteristic disease features including vascular, inflammatory, and fibrotic complications indicating their role in SSc pathogenesis. Therefore, the impact of anti-AT_1_R and anti-ET_A_R Abs on initiation of inflammation and fibrosis was analyzed.

**Methods:**

Anti-AT_1_R and anti-ET_A_R Ab-positive immunoglobulin G (IgG) from SSc patients (SSc-IgG) was used for experiments. Healthy donor IgG served as a normal control, and AT_1_R and ET_A_R activation was inhibited by antagonists. Protein expression was measured with ELISA, mRNA expression with real time-PCR, endothelial repair with a scratch assay, and collagen expression with immunocytochemistry. Transendothelial neutrophil migration was measured with a culture insert system, and neutrophil ROS activation with immunofluorescence. Neutrophils in bronchoalveolar lavage fluids (BALFs) were analyzed microscopically after passive transfer of SSc-IgG or NC-IgG into naïve C57BL/6J mice. KC plasma levels were quantified by a suspension array system. Histologic analyses were performed by using light microscopy.

**Results:**

Anti-AT_1_R and anti-ET_A_R Ab-positive SSc-IgG induced activation of human microvascular endothelial cells (HMEC-1). Elevated protein and mRNA levels of the proinflammatory chemokine interleukin-8 (IL-8, CXCL8) and elevated mRNA levels of the vascular cell adhesion molecule-1 (VCAM-1) were induced in HMEC-1. Furthermore, activation of HMEC-1 with SSc-IgG increased neutrophil migration through an endothelial cell layer and activation of reactive oxygen species (ROS). SSc-IgG decreased HMEC-1 wound repair and induced type I collagen production in healthy donor skin fibroblasts. Effects of migration, wound repair, and collagen expression were dependent on the Ab-levels. Passive transfer of anti-AT_1_R and anti-ET_A_R Ab-positive SSc-IgG into naïve C57BL/6J mice increased neutrophil BALF counts. In parallel, increased levels of the murine functional IL-8 homologue, chemokine KC, were found in the plasma of SSc-IgG-treated mice as well as structural alterations of the lungs.

**Conclusions:**

We conclude that angiotensin and endothelin-receptor activation via anti-AT_1_R and anti-ET_A_R Abs mediate pathogenic effects, indicating their contribution to pathogenesis of SSc. Therefore, anti-AT_1_R and anti-ET_A_R Abs could provide novel targets for therapeutic intervention in the treatment of SSc.

## Introduction

Systemic sclerosis (SSc) is an autoimmune disorder with severe clinical manifestations, high mortality, and limited therapeutic options. Autoimmunity, vasculopathy, and fibrosis are hallmarks of the disease [1,2]. So far, mechanisms by which these hallmarks may be linked together are not well understood. Recent work from our group has shown that anti-AT_1_R and anti-ET_A_R Abs are present in SSc [3], and that elevated Ab levels in sera are correlated with major disease manifestations, emphasizing their potential role in SSc pathogenesis. It is well established that microvascular damage, featuring endothelial cell dysfunction and perivascular infiltrates, is a key event in SSc pathogenesis appearing early in the course of the disease and preceding fibrosis [4-6]. Inflammation also is a crucial event in SSc development and is reflected by abnormal chemokine and cytokine levels in sera and BALF [7-9], as well as by inflammatory infiltrates [2,4]. Of note are elevated levels of IL-8, both in sera and in BALF [7,9,10]. Furthermore, the latter were connected to neutrophilic alveolitis in SSc-related interstitial lung disease [7,11], demonstrating a link between increased IL-8 levels and neutrophil accumulation.

Progressive fibrosis is characterized by amplified production of extracellular matrix (ECM) components including increased collagen synthesis by fibroblasts. SSc skin fibroblasts have been demonstrated to produce higher amounts of collagen when compared with skin fibroblasts from healthy donors [12,13]. Similarly, increased collagen expression was found in an animal model of SSc [14].

Last, an increased activation of the angiotensin and endothelin axis has been reported in SSc [15-17]. Accordingly, we reasoned that anti-AT_1_R and anti-ET_A_R Abs could directly contribute to the initiation of inflammation and fibrosis *in vitro* and *in vivo* by activation of endothelial cells, fibroblasts, and neutrophils and thus contribute to the key pathogenic manifestations of SSc. The objective of this study was to analyze the impact of functional anti-AT_1_R and anti-ET_A_R Abs on inflammatory and fibrotic events to help understand their role in disease pathogenesis.

## Methods

### Reagents

All reagents were purchased from Sigma Aldrich (Germany), if not otherwise stated.

### Ethical admission for patient sample collection and performance of animal experiments

Serum was collected from venous blood after written informed consent and local ethics committee approval (EA1/013/705). Healthy donor skin was obtained by biopsy after written informed consent and approval by University Hospital Charité ethics committee (EA1/168/06). C57BL/6J mice were obtained from Charles River (Sulzfeld, Germany). Experiments were performed according to institutional and federal guidelines (Landesamt für Gesundheit und Soziales, Berlin, Germany).

### Patients and healthy control donors

SSc patients with diffuse or limited SSc were classified according to LeRoy and ACR criteria [1,18]. Patients with established vasculopathy and/or fibrosis, including pulmonary arterial hypertension (PAH), lung and skin fibrosis, were chosen for IgG isolation that subsequently was used for experiments. Healthy control subjects served as negative controls. Identical IgG processing was used for SSc patients and healthy donors for serum collection and IgG isolation. For functional assays, individual IgG samples were used that were isolated from one serum sample as described later, followed by measurement of anti-AT_1_R and anti-ET_A_R Abs levels of each sample, as previously reported [3]. For animal experiments, the same methods were used, except that IgG was isolated from a pool of sera (from several patients or healthy donors) to provide enough material. Detailed patient and healthy donor control characteristics are summarized in Table 1.

**Table 1:** Patient and healthy donor characteristics

**Parameter**	**SSc patients (*n *** **= 33)**	**Healthy donors (*n *** **= 13)**
Mean age, years (SD)	55 (13)	43 (7)
Females/males, *n* (%)	24/9 (73/27)	9/4 (69/31)
Mean anti-AT_1_R Abs, units (SD)	16 (8)	9 (4)
Mean anti-ET_A_R Abs, units (SD)	15 (10)	6 (4)
Diffuse cutaneous form, limited cutaneous form, other, *n* (%)	21 (64), 10 (30), 2 (6)	n.a.
Scl 70 positive, *n* (%), n.d. *n* (%)	13 (39), 3 (90)	n.a.
Anti-centromere positive, *n* (%), n.d. *n* (%)	4 (12), 4 (12)	n.a.
Duration since Raynaud phenomenon, years (±SD)	10 (11)	n.a.
Duration since skin-involvement onset, years (±SD)	9 (7)	n.a.
Duration since internal organ onset, years (±SD)	8 (7)	n.a.
mRSS median (IQR)	8 (4-18)	n.a.
Pulmonary arterial hypertension, *n* (%)^a^	14 (42)	n.a.
Lung fibrosis, *n* (%)^b^	18 (55)	n.a.
Mean DLCO% (SD)	50 (20)	n.a.
Mean FVC% (SD)	85 (18)	n.a.
**Animal experiments**	**SSc patients (n = 14)**	**Healthy donors (n = 15)**
Mean age, years (SD)	57 (14)	47 (8)
Females/males, *n* (%)	11/3 (79/21)	12/3 (80/20)
Mean anti-AT_1_R Abs, units (SD)	18 (10)	6 (4)
Mean anti-ET_A_R Abs, units (SD)	17 (10)	4 (3)
Diffuse cutaneous form, limited cutaneous form, other, *n* (%)	10 (71), 3 (21), 0 (0)	n.a.
Scl 70 positive, *n* (%), n.d. *n* (%)	10 (71), 1 (7)	n.a.
Anti-centromere positive, *n* (%), n.d. *n* (%)	2 (14), 3 (21)	n.a.
Duration since Raynaud phenomenon, years (±SD)	11 (8)	n.a.
Duration since skin-involvement onset, years (±SD)	10 (7)	n.a.
Duration since internal-organ onset, years (±SD)	10 (9)	n.a.
mRSS median (IQR)	8.5 (5.3-11)	n.a.
Pulmonary arterial hypertension, *n* (%)^a^	8 (57)	n.a.
Lung fibrosis, *n* (%)^b^	8 (57)	n.a.
Mean DLCO% (SD)	42 (18)	n.a.
Mean FVC% (SD)	77 (19)	n.a.

n.d., not defined; n.a., not applicable.

^a^By >35 mm Hg sPAP in echocardiography and/or 25 mm Hg mPAP in right-heart catheterization.

^b^By HR-CT or x-ray.

### Isolation of IgG and detection of anti-AT_1_R and anti-ET_A_R Abs

IgG was isolated by protein-G sepharose chromatography in 20 m*M* phosphate buffer pH 7.0. IgG was eluted with 0.1 *M* glycine/HCl, pH 2.7, and pH was neutralized with 1 *M* Tris/HCl, pH 9.0. Eluted IgG was dialyzed against PBS, and absorbance was measured at 280 nm (Emax, Molecular Devices, USA). Anti-AT_1_R and anti-ET_A_R Abs were detected in purified IgG in cooperation with CellTrend GmbH (Germany) with a commercially available solid-phase assay (One Lambda, Inc., USA), as described previously [3].

### Cultivation and treatment of cells

Human microvascular endothelial cells-1 (HMEC-1s) were serum-starved before all experiments in endothelial cell medium with IgG-free fetal calf serum (FCS) 0.5%, penicillin, 100 U/ml; streptomycin, 100 μg/ml, hydrocortisone, 25 μ*M*, epidermal growth factor 0.01 μg/ml, and L-glutamine, 10 m*M*. Cells were incubated in a humidified atmosphere at 5% CO_2_ and 37°C. Human fibroblasts were isolated from healthy donor skin. Dermis was removed by dispase (4 mg/ml), and epidermis was digested with collagenase type 1A (1 mg/ml). Fibroblasts were cultivated in DMEM with IgG-free FCS, 10%, penicillin, 100 U/ml, streptomycin, 100 μg/ml, and amphotericin B, 2.5 μg/ml. For all experiments, passages three to eight were serum-starved before experiments in DMEM with IgG-free FCS 1%, penicillin 100 U/ml, streptomycin 100 μg/ml and amphotericin B 2.5 μg/ml. For transendothelial migration, neutrophils were freshly isolated from healthy donor blood, as described [19]. Isolated neutrophils were added to phosphate-buffered saline (PBS)/IgG-free FCS 10%, and their migration capacity was assessed with transwell culture inserts, as described later. All reagents were purchased from PAA Laboratories (Germany) and Invitrogen (Europe). For all experiments that included receptor antagonism, inhibitors were added to cell cultures 18 and 3 hours before IgG treatment. The most effective antagonist concentration was determined in serial experiments.

For individual receptor antagonism, AT_1_R was inhibited by valsartan, and the ET_A_R by the selective inhibitor sitaxentan. In parallel, the ET_A_R was inhibited by the dual antagonist bosentan. All antagonists were used at 10^–5^*M* concentration, as described earlier [16,20]. Antagonists were also applied simultaneously by combination of valsartan and sitaxentan (each at 10^–7^*M*), or valsartan and bosentan (at 10^–5^*M* and 5 × 10^–7^*M*, respectively). For inactivation of NF-κB, tosyl-L-phenylalanine chlormethyl ketone (TPCK) was used (3 × 10^–6^*M*) 30 minutes before IgG treatment. All antagonists tested nontoxic, individually or simultaneously, in a cell-viability test (WST-8; Dojindo, Japan). Dose-dependent experiments were performed for IL-8 protein expression, as described later (range of 0.125 mg/ml to 1.5 mg/ml IgG). An IgG concentration of 1 mg/ml was used in all experiments described. Of note, a similar IgG concentration was used previously [21]. Angiotensin II (Ang II) and endothelin-1 (ET-1) were used at 10^–6^*M* and 10^–8^*M* concentrations, respectively, and incubation times were the same as for IgG [16,20].

### Scratch assay

For analysis of endothelial repair, uniform scratches were made by 1-ml pipette tip in confluent HMEC-1 layers, as described [22]. HMEC-1 cells were allowed to migrate into scratch areas to close wounds for 24 hours in the presence of SSc-IgG or NC-IgG. Cells were fixed in 96% ethanol, stained with hematoxylin and eosin (Merck, Germany), and light-microscopy pictures were taken (Leica DMIL LED, LAS-EZ 2.0, Germany). Scratch areas were semiquantified with ImageJ software by measuring relative scratch areas.

### RNA, cDNA, and real-time PCR

RNA was isolated from HMEC-1 48 hours after IgG treatment by NucleoSpin RNA II (Macherey-Nagel, Germany), and cDNA was generated by M-MLV reverse transcriptase (Promega, Germany), each according to manufacturer’s instructions. Real-Time PCR reactions contained 5 μl of cDNA, 0.25 m*M* dNTP (Bioline, Germany), 12 μg/ml bovine serum albumin, 1 × SYBR Green-I (Molecular Probes, Germany), 1 U Immolase (Bioline), 500 m*M* TRIS pH 8.8, 6 m*M* MgCl_2_, 0.5 nmol/ml primer mix (TIB MOLBIOL, Germany), and were performed in MX3000P cycler (Stratagene, Europe). Primers were designed by Primer3 [23]. IL-8 forward 5′CAA-GAG-CCA-GGA-AGA-AAC-CA3′, reverse 5′ACT-CCT-TGG-CAA-AAC-TGC-AC3′. VCAM-1 forward 5′AAG-ATG-GTC-GTG-ATC-CTT-GG3′, reverse 5′GGT-GCT-GCA-AGT-CAA-TGA-GA3′. Eukaryotic translation elongation factor 1-α 1 (EEF1A1) was used as housekeeping gene, forward 5′GTT-GAT-ATG-GTT-CCT-GGC-AAG-C3′, reverse 5′GCC-AGC-TCC-AGC-AGC-CTT-C3′. Samples were analyzed with MxPro-Mx3005P (Stratagene), and expression levels were normalized to the housekeeping gene.

### Detection of IL-8 protein

HMEC-1 supernatants were collected 48 hours after IgG treatment. IL-8 was measured with sandwich ELISA. The coating antibody and the biotinylated detection antibody were obtained as a matched-pairs kit from ImmunoTools (Germany). Recombinant human IL-8 was obtained from Biolegend (Germany) to generate a standard curve and streptavidin conjugated HRP was obtained from Biolegend (Germany). All reagents were used according to manufacturer’s instructions. Absorbance was measured at 450 nm by using the E_max_ microplate reader (Molecular Devices). Data were analyzed by SoftMax Pro v5 (Molecular Devices).

### Transendothelial neutrophil migration and measurement of neutrophil derived ROS

Supernatants from IgG-treated HMEC-1 (SSc-IgG or NC-IgG, 48 hours) were placed in multiwell TM 24 plates followed by confluent HMEC-1 on transwell culture inserts (3 μ*M* pore size, all Becton Dickinson). Anti-IL-8 antibody (AB-208-NA; R&D Systems, Germany) was added to supernatants (10 μg/ml) 30 minutes before migration was assessed. Neutrophils were freshly isolated from healthy donor blood, as described earlier and added to inserts (2 × 10^6^ cells/insert). Migrated cells were counted automatically after 4 hours (CASY; Schärfe Systems, Germany). In parallel, neutrophils were isolated as described earlier and were loaded with DCFH-DA (2′,7′-dichlorofluorescin diacetate, 25 μ*M* in PBS/IgG-free FCS 1%) in a humidified atmosphere at 5% CO_2_ and 37°C. Loaded neutrophils were washed with 37°C warm PBS, and 1 × 10^5^ neutrophils were added to 200 μl of HMEC-1 supernatants treated with IgG in a 96-well round-bottom cell-culture plate. After 30 minutes at 5% CO_2_ and 37°C, cells were washed with PBS and fixed in 2% paraformaldehyde for 30 minutes at 4°C, washed again, and taken up in 150 μl PBS. Cells were transferred into a white 96-well plate suitable for colorimetric analysis (F96 MicroWell; Nunc, Germany), and generation of reactive oxygen species (ROS) was analyzed at an excitation wavelength of 485 nm and at an emission wavelength of 538 nm by using a fluorescence reader (Fluoroskan Ascent, Thermo Labsystems, Germany).

### Collagen detection

Confluent fibroblasts isolated from healthy donor skin were grown on glass chamber slides (Iwaki, Japan) and treated with SSc-IgG or NC-IgG for 5 days for maximum collagen expression. Cells were fixed in paraformaldehyde (2%) and triton X-100 (0.1%). Type I collagen immunocytochemistry was performed by using a monoclonal primary antibody reactive to human collagen protein 1A (sc-59772; Santa Cruz, Santa Cruz, CA, USA) and a secondary Cy3-conjugated antibody (C2181; Sigma-Aldrich). Nucleic DNA was stained with 4′,6-diamidino-2-phenylindole dihydrochloride (DAPI). Monochrome fluorescence pictures were taken (Axioplan; Carl Zeiss MicroImaging GmbH, Germany) at identical illumination times, and fluorescence intensity signals were analyzed relative to cell number by ImageJ software (NIH).

### Antibody transfer into naïve mice

Female 7-week-old C57BL/6J mice (*n* = 7/group) were maintained under specific pathogen-free conditions and received intravenously endotoxin-free pooled NC-IgG or SSc-IgG (800 μg, dissolved in 100 μl NaCl 0.9%) at day 0, as previously described for anti-AT_1_R Abs from preeclampsia patients to study systemic events [24]. Pooled IgG fractions were tested for anti-AT_1_R and anti-ET_A_R Abs, as described earlier. NC-IgG fraction of low Ab levels (anti-AT_1_R Abs units of 3.85 and anti-ET_A_R Abs units of 2.5) and SSc-IgG fraction of high Ab levels (anti-AT_1_R Abs units of 21.8 and anti-ET_A_R Abs units of 17.91) were used for transfer. At day 7, mice were killed, blood was harvested, and BALF of the right lung was collected by using 2 × 650 μl PBS. Leukocytes were counted and differentiated by means of microscopic analysis in a blinded fashion (800 cells counted/individually). KC plasma levels were quantified by Bio-Plex array according to the manufacturer’s guide. For repeated IgG treatment, female 8-week-old C57BL/6J mice (*n* = 7/group) were maintained as described earlier. Mice were treated with NC-IgG or SSc-IgG intravenously with pooled IgG at day 1, day 17, day 30, and 7 days before analysis, for a total of 100 days (200 μg, dissolved in 100 μl NaCl 0.9%). Histology analysis of the lungs was performed by paraffin embedding, hematoxylin and eosin (H&E) staining, and light microscopy (Axioplan 2; Carl Zeiss MicroImaging GmbH).

### Statistical analysis

Results were analyzed with GraphPad Prism software (version 5.02) by using Mann-Whitney *U* test (NC-IgG compared with SSc-IgG) and Wilcoxon signed-rank test (SSc-IgG compared with SSc-IgG with blockers). Correlation analyses were performed by nonparametric correlation (Spearman) and linear-regression correlation. **P < 0.01 and *P < 0.05.

## Results

### Induction of IL-8 expression and release by endothelial cells

**Figure 1:**
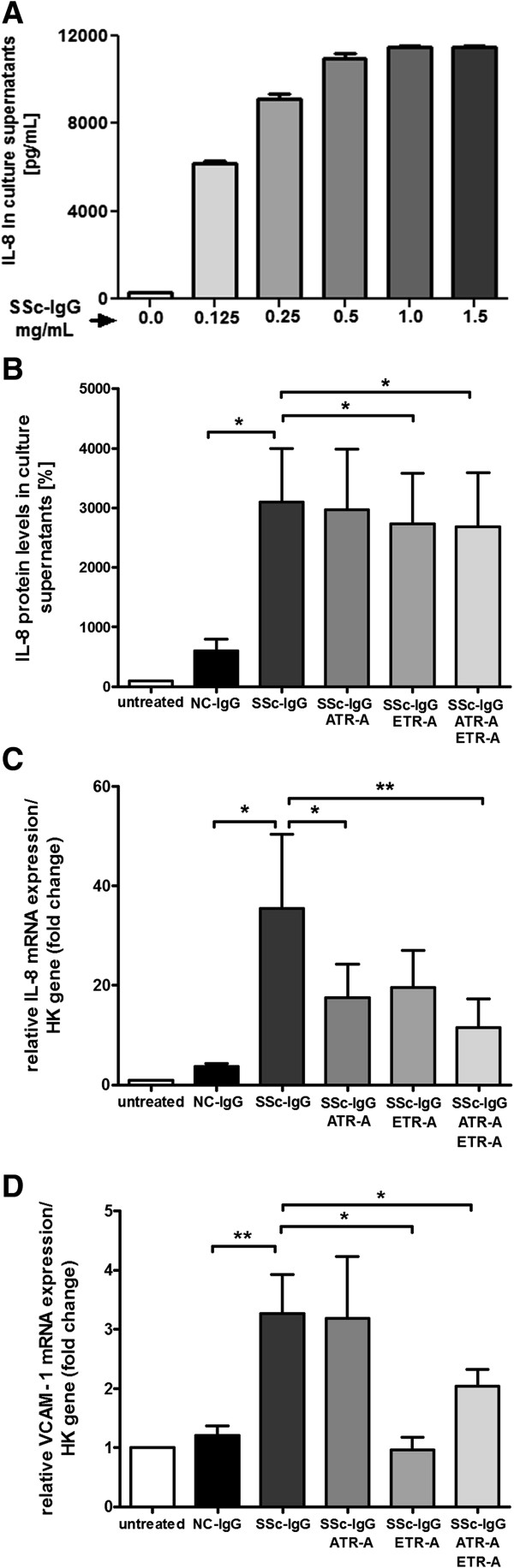
**Activation of HMEC-1 by anti-AT**_**1**_**R and anti-ET**_**A**_**R Ab-positive SSc-IgG on protein and mRNA levels*. *(A)** Dose-dependent IL-8 secretion on different doses of anti-AT_1_R and anti-ET_A_R Ab-positive SSc-IgG. Same treatment with NC-IgG failed to demonstrate a dose-dependent IL-8 secretion pattern. **(B)** Significant increase in IL-8 secretion with SSc-IgG versus NC-IgG and decrease with receptor antagonism (NC-IgG, *n* = 9, SSc-IgG *n* = 13, *P* < 0.05). **(C)** Increase in IL-8 mRNA levels by SSc-IgG versus NC-IgG (NC-IgG, *n* = 7, SSc-IgG *n* = 18, *P* < 0.05). Receptor antagonism leads to inhibition of IL-8 secretion, as indicated (SSc-IgG, *n* = 13, *P* < 0.05, *P* < 0.01). **(D)** Increase in VCAM-1 mRNA levels with SSc-IgG compared with NC-IgG (NC-IgG, *n* = 5, and SSc-IgG, *n* = 6; *P* < 0.01) and inhibition by receptor antagonism as indicated (*P* < 0.05). Mean and SEM, **P < 0.01, and *P < 0.05.

Analysis of HMEC-1 activation by anti-AT_1_R and anti-ET_A_R Abs-positive SSc-IgG showed a secretion of the proinflammatory and profibrotic chemokine IL-8 into culture supernatants. A dose-dependent pattern of IL-8 protein levels was found in HMEC-1 cells on stimulation with 0.125 mg/ml to 1.5 mg/ml SSc-IgG, with the highest response between 0.5 and 1.5 mg/ml IgG (Figure 1A), that was not present with NC-IgG. A comparison of SSc-IgG versus NC-IgG treatment revealed increased IL-8 levels with SSc-IgG treatment, with high variability in individual IgG samples (*P* < 0.05; Figure 1B). Increased IL-8 protein levels were reduced by individual as well as simultaneous receptor antagonism (ETR-A and ATR-A/ETR-A, each *P* < 0.05; Figure 1B). We further analyzed IL-8 expression on an mRNA level and found a significant increase in SSc-IgG- over NC-IgG-treated cells (*P* < 0.05; Figure 1C). Elevated IL-8 mRNA levels were reduced by receptor antagonism, as indicated (ATR-A and AT1R-A/ETR-A; *P* < 0.05 and *P* < 0.01, respectively, Figure 1C). Similarly, significantly increased mRNA levels of VCAM-1 were induced by SSc-IgG treatment compared with NC-IgG (*P* < 0.01; Figure 1D) and reduced by receptor antagonism (ETR-A and ATR-A/ETR-A, each *P* < 0.05; Figure 1D).

### Induction of IL-8 and Ab-level-dependent neutrophil transendothelial migration and ROS activation

**Figure 2:**
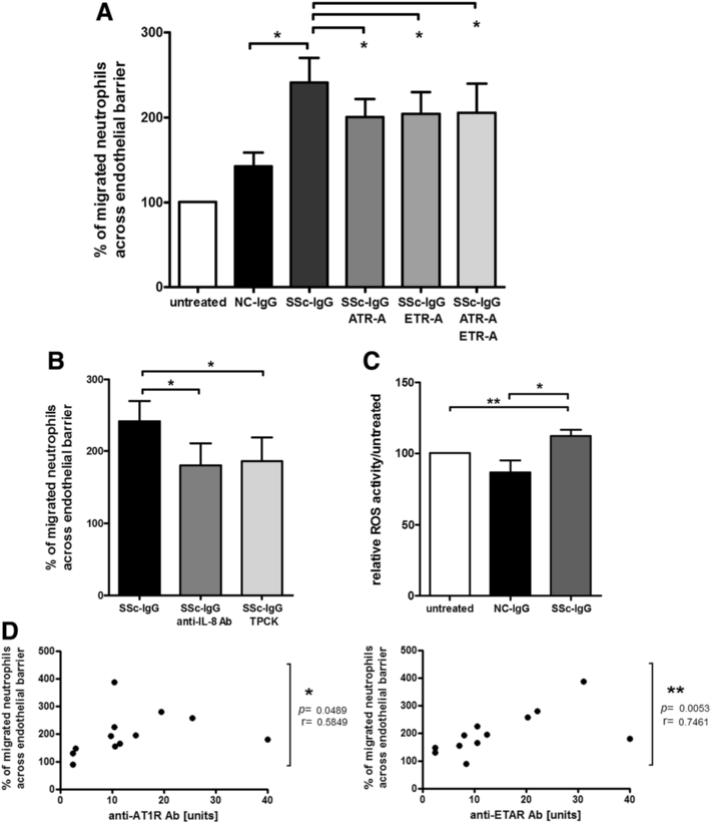
**Neutrophil migration and ROS activation induced by anti-AT**_**1**_**R and anti-ET**_**A**_**R Ab-positive SSc-IgG.** Supernatants of HMEC-1 conditioned with anti-AT_1_R and anti-ET_A_R Ab-positive SSc-IgG induce activation and recruitment of neutrophils, measured by transendothelial migration and ROS generation. **(A)** Significantly increased transendothelial neutrophil migration with SSc-IgG-conditioned supernatants compared with NC-IgG and decrease in samples with receptor antagonism, as indicated (NC-IgG, *n* = 5, SSc-IgG, *n* = 7, *P* < 0.05). **(B)** Neutrophil migration is induced by IL-8 in supernatants shown by IL-8 neutralization (anti-IL-8 Ab) and NF-κB inactivation (TPCK) (SSc-IgG *n* = 7, *P* < 0.05). **(C)** Increased ROS activation in neutrophils treated with SSc-IgG conditioned HMEC-1 supernatants compared with NC-IgG or untreated (NC-IgG, *n* = 8, SSc-IgG, *n* = 12, P < 0.05; *P* < 0.01). Mean and SEM. **P < 0.01 and *P < 0.05. **(D)** Neutrophil migration shows a positive correlation to anti-AT1R Abs and anti-ETAR Abs in the IgG samples used (NC-IgG, *n* = 5; SSc-IgG, *n* = 7; total, *n* =12). Spearman correlation.

Neutrophil recruitment and migration were analyzed by transendothelial migration and ROS generation. Supernatants of SSc-IgG-treated HMEC-1 increased healthy donor neutrophil migration through an endothelial cell layer compared with supernatants of NC-IgG-treated HMEC-1 (*P* < 0.05; Figure 2A). Neutrophil migration toward supernatants was significantly reduced by receptor inhibitors (ATR-A, ETR-A, and ATR-A/ETR-A; all *P* < 0.05, Figure 2A). Addition of an IL-8-neutralizing antibody to SSc-IgG treated samples, as well as addition of NF-κB inactivator TPCK, significantly decreased neutrophil transendothelial migration (each *P* < 0.05, Figure 2B). Finally, SSc-IgG-conditioned HMEC-1 supernatants significantly increased generation of ROS in healthy donor neutrophils compared with NC-IgG conditioned supernatants or untreated controls (*P* < 0.05 and *P* < 0.01, respectively, Figure 2C). Statistical analyses revealed a significant correlation between neutrophil migration and anti-AT_1_R and anti-ET_A_R Ab levels (*r* = 0.5849 and *r* = 0.7461, respectively, P < 0.05 and *P* < 0.01, respectively, Figure 2D).

### Influence on endothelial-repair function

**Figure 3:**
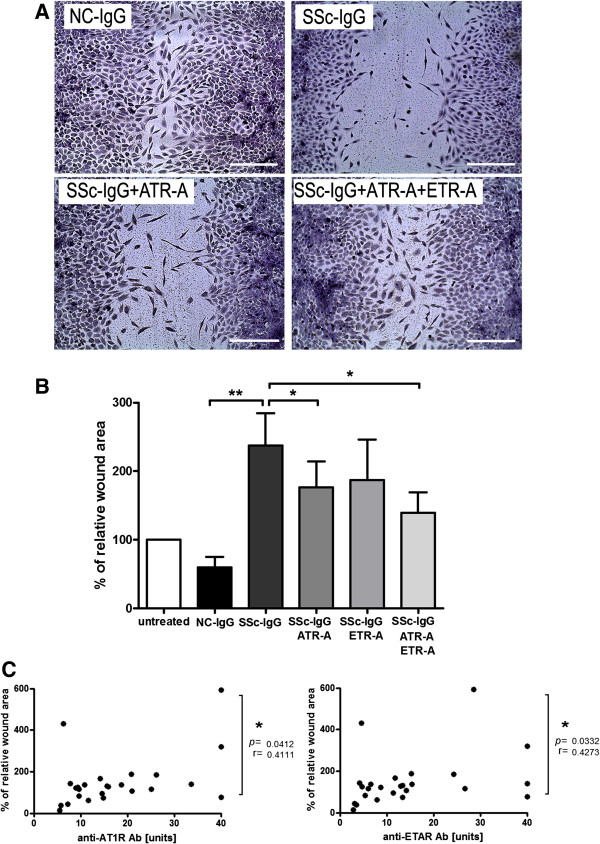
**Diminished endothelial repair by treatment with anti-AT**_**1**_**R and anti-ET**_**A**_**R Ab-positive SSc-IgG*.*** HMEC-1 treated with anti-AT_1_R and anti-ET_A_R Ab-positive SSc-IgG reduce wound areas measured by a scratch assay. **(A)** Representative pictures of NC-IgG and of SSc-IgG-treated cells and indicated inhibitors. **(B)** After 24 hours, wound areas are significantly greater in SSc-IgG versus NC-IgG treatment (NC-IgG, *n* = 6, SSc-IgG *n* = 6, *P* < 0.01) and are significantly reduced by antagonists, as indicated (*P* < 0.05). Bar indicates 250 micron. Independent experiments were performed at least twice. Mean and SEM, **P < 0.01 and *P < 0.05. **(C)** Wound area, as measurement of endothelial repair, shows a correlation to levels of anti-AT1R Abs and anti-ETAR Abs (NC-IgG, *n* = 11; SSc-IgG, *n* = 14; total *n* = 25). Spearman correlation.

As we observed endothelial cell activation and neutrophil recruitment, we also analyzed the influence of anti-AT_1_R and anti-ET_A_R Abs on endothelial-repair function. Artificially generated wounds in HMEC-1 layers were analyzed in a scratch assay. Reduced cell-layer repair was reflected by a larger wound area in HMEC-1 treated with SSc-IgG compared with NC-IgG (*P* < 0.01, Figure 3A and B). Individual and simultaneous receptor antagonism improved endothelial repair of SSc-IgG-treated cells with significant scratch-area reduction (ATR-A, ATR-A/ETR-A; all *P* < 0.05, Figure 3B). Correlation analyses showed a significant relation between impaired endothelial repair, reflected by the wound area and anti-AT_1_R and anti-ET_A_R Ab levels (*r* = 0.4111 and *r* = 0.4273, respectively; *P* < 0.05, Figure 3C).

### Induction of collagen expression in healthy donor-skin fibroblasts

**Figure 4:**
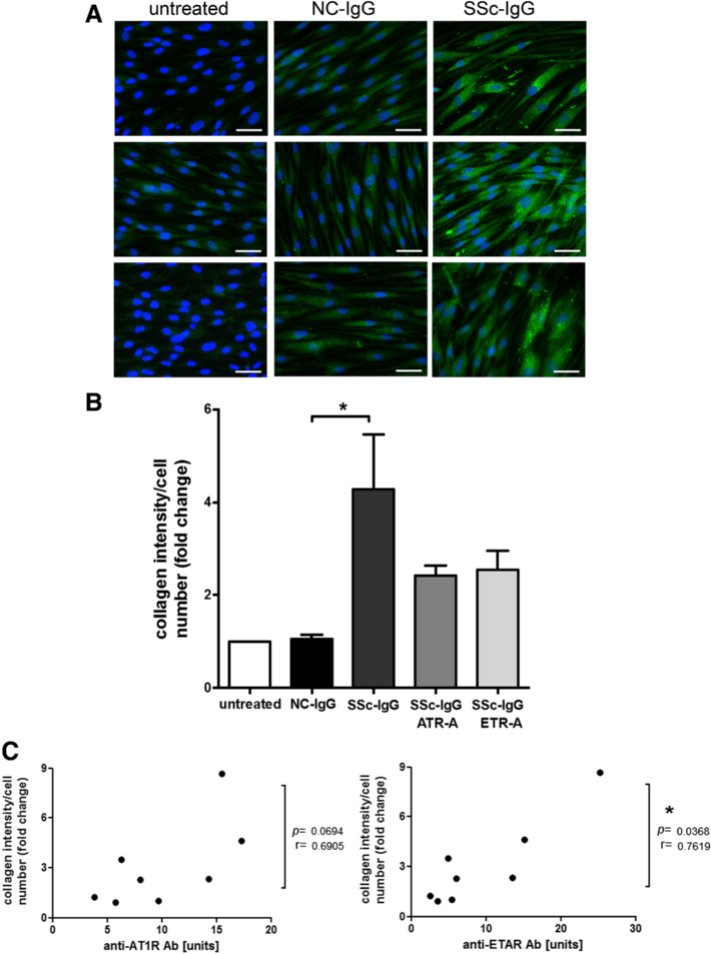
**Induction of collagen expression in fibroblasts by anti-AT**_**1**_**R and anti-ET**_**A**_**R Ab-positive SSc-IgG*.*** Skin fibroblasts of healthy donors increase expression of type I collagen with anti-AT_1_R and anti-ET_A_R Ab positive SSc-IgG. **(A)** Expression of type I collagen analyzed with immunocytochemistry. Collagen expression is shown in green, and nucleic acid DAPI stain, in blue. Bar indicates 50 μm; shown are representative pictures. **(B)** Significant increase in type I collagen with SSc-IgG versus NC-IgG (NC-IgG *n* = 4 and SSc-IgG *n* = 5, *P* < 0.05). Relative fluorescence intensity was analyzed and normalized to cell number for each sample. Mean and SEM, *P < 0.05. **(C)** Collagen expression (type I collagen) shows a significant correlation to anti-ETAR Ab levels and a trend to anti-AT1R Ab levels (NC-IgG, *n* = 3;, SSc-IgG, *n* = 5;, total *n* = 8; *P* < 0.05). Spearman correlation.

Because profibrotic events could be induced by anti-AT_1_R and anti-ET_A_R Abs-positive SSc-IgG in HMEC-1 and neutrophil recruitment by IL-8, we additionally investigated profibrotic effects on fibroblasts as major collagen-expressing cells. Human fibroblasts were isolated from healthy donor skin, and expression of type I collagen was measured by immunocytochemistry on treatment with anti-AT_1_R and anti-ET_A_R Abs-positive SSc-IgG or NC-IgG. Increased type I collagen expression was found with SSc-IgG treatment compared with NC-IgG (Figure 4A). Measurement of collagen intensity relative to cell number showed significantly increased collagen content in SSc-IgG treated cells over NC-IgG-treated cells (*P* < 0.05, Figure 4B). Antagonism of ATR-A and ETR-A resulted in a marked, not significant reduction of collagen, due to high variability in the tested samples. Statistical tests demonstrated a significant correlation between collagen induction and anti-ETAR Ab levels, whereas only a marked tendency was observed to anti-AT1R Ab levels (*r* = 0.7619 and *r* = 0.6905, respectively; P < 0.05 and *P* = 0.0694, respectively, Figure 4C).

### Induction of pulmonary neutrophil recruitment, increased plasma levels of murine IL-8 analogue KC, and structural alterations in lungs of naïve C57BL/6J mice

**Figure 5:**
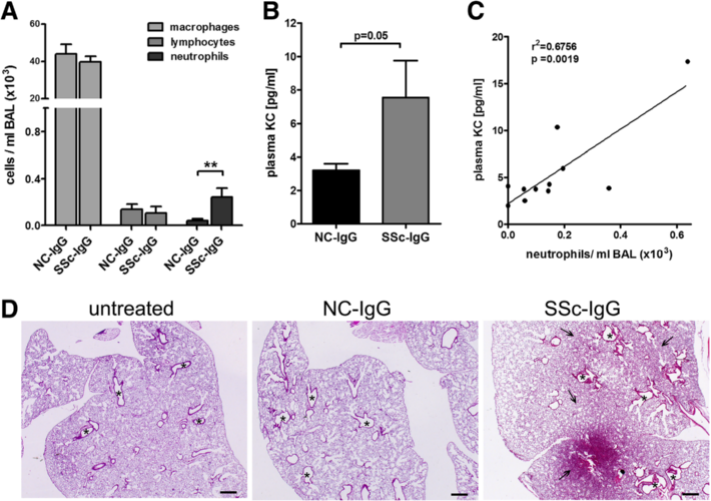
**Systemic effects in naïve mice induced by anti-AT**_**1**_**R and anti-ET**_**A**_**R Ab-positive SSc-IgG*.*** Anti-AT_1_R and anti-ET_A_R Ab-positive SSc-IgG transfer increases neutrophil recruitment and KC levels (murine IL-8 analogue) in mice. **(A)** Significantly increased neutrophil counts detected in BALF of mice treated with SSc-IgG compared with NC-IgG (*n* = 7; P < 0.01). **(B)** KC plasma levels in SSc-IgG-treated mice as compared with NC-IgG-treated mice are increased by trend (NC-IgG, *n* = 5; SSc-IgG, *n* = 6; *P* = 0.05). **(C)** KC plasma levels significantly correlate with neutrophil counts in BALF (NC-IgG *n* = 5, SSc-IgG, *n* = 6, *r*^*2*^ = 0.6756, *P* = 0.0019, linear regression correlation). Mean and SEM, **P < 0.01. **(D)** Repeated treatment with IgG results in marked alterations of the lung structure of SSc-IgG-treated mice compared with NC-IgG or untreated mice. Shown are representative light-microscopy pictures, H&E staining, ×12.5 magnification; bar indicates 500 μm. Asterisks indicate examples of airway vessels; arrows indicate elevated cell density in interstitial tissue.

To analyze systemic effects of anti-AT_1_R and anti-ET_A_R Abs *in vivo*, naïve C57BL/6J mice were subjected to passive transfer of pooled SSc-IgG or pooled NC-IgG, as previously described [24]. Seven days after the transfer, increased numbers of neutrophils were found in BALF of SSc-IgG-treated mice as compared with NC-IgG-treated mice (*P* < 0.01, Figure 5A), whereas no differences were observed for macrophages or lymphocytes. Eosinophils were not detectable. Structural alteration of the lungs were not observed by a single IgG-treatment.

However, plasma levels of the murine IL-8 analogue KC were found to be increased after SSc-IgG treatment compared with NC-IgG treatment (*P* = 0.05, Figure 5B). Moreover, correlation analysis of KC plasma levels and BALF neutrophil counts showed a strong positive correlation (*r*^*2*^ = 0.6756 and P = 0.0019, Figure 5C). Repeated IgG treatment resulted in profound structural alteration of the lungs, including increased cellular density and interstitial cellular infiltrations in mice treated with SSc-IgG compared with NC-IgG or untreated mice (Figure 5D).

## Discussion

The purpose of this study was to analyze the impact of anti-AT_1_R and anti-ET_A_R Abs on the induction of vascular inflammation and fibrosis, the key features of SSc. The presence of elevated anti-AT_1_R and anti-ET_A_R Ab levels in sera of SSc patients correlates with an increased risk for the development of lung fibrosis, pulmonary arterial hypertension (PAH), as well as with mortality, as demonstrated previously [3]. Furthermore, these Abs induced the expression of transforming growth factor-β (TGF-β) in HMEC-1s, suggesting a potential involvement in fibrosis [3]. Therefore, we sought to analyze the actions of anti-AT_1_R and anti-ET_A_R Abs with a focus on inflammation and fibrosis.

Here, we demonstrate that IgG samples from SSc patients positive for anti-AT_1_R and anti-ET_A_R Abs induce proinflammatory and fibrotic events in endothelial cells and healthy donor fibroblasts via angiotensin and endothelin-receptor activation. Besides the activation of fibroblasts, possible pathogenic effects mediated by SSc-IgG were reflected by endothelial dysfunction, expression of IL-8, and increased neutrophil migration into target tissues.

Involvement of both the angiotensin and the endothelin systems in SSc pathogenesis has been demonstrated previously: Elevated serum levels of Ang II and ET-1 in SSc patients were reported, as well as increased ET-1 levels in SSc lung fibrotic tissue, indicating their central role in SSc pathogenesis [15,25,26]. Also, a link between angiotensin and endothelin-receptor activation and fibrosis, perhaps the most prominent feature of SSc, was also suggested in the literature [26-28]. Activation of AT_1_R by anti-AT_1_R Abs was reported previously for preeclampsia and renal-allograft rejection [24,29]. Overexpression of extracellular matrix components (ECM), of which collagen represents an important element, is a key aspect in fibrosis development [12,14]. Angiotensin- and endothelin-mediated collagen expression was demonstrated [17,27]. Accordingly, we found increased expression of collagen in healthy donor dermal fibroblasts after exposure to anti-AT_1_R and anti-ET_A_R Ab-positive SSc-IgG. The intensity of collagen expression was significantly dependent on levels of anti-ET_A_R Abs, showing an Ab-dependent effect.

Besides the importance of fibrosis in SSc, many studies propose that microvascular damage and inflammation can precede fibrosis [4,30]. Additionally, autoimmune-mediated damage to endothelial cells has been demonstrated to cause endothelial dysfunction [31], which can lead to vessel leaks and lymphocyte infiltration [4,32]. In this regard, anti-AT_1_R and anti-ET_A_R Abs induced microvascular endothelial cell (HMEC-1) dysfunction after exposure to positive SSc-IgG, resulting in reduced endothelial repair in an Ab-level-dependent manner. Moreover, endothelial dysfunction was further reflected by VCAM-1 expression on endothelial cells. Recently, the concept of vascular leak was proposed to be a central feature of SSc pathogenesis, highlighting the importance of changes in the microvasculature in disease progression [33]. Our findings indicate a general disturbance of endothelial functions by SSc-IgG *in vitro,* which could probably also occur *in vivo*. However, this hypothesis must be tested in more detail in future experiments.

Furthermore, expression of the chemokine IL-8 with proinflammatory and profibrotic properties has been reported to be increased in sera, BALF, and fibroblasts in SSc [7,8,10]. Accordingly, we found increased mRNA and protein levels of IL-8 in HMEC-1 cells after exposure to anti-AT_1_R and anti-ET_A_R Ab positive SSc-IgG. In line with this, the murine IL-8 functional homolog KC was found to be increased in plasma of naïve mice treated with SSc-IgG. Given the chemotactic abilities of IL-8, we congruously found increased neutrophil transendothelial migration toward supernatants of SSc-IgG-activated endothelial cells, which was dependent on IL-8. Increased neutrophil counts were also detected *in vivo* in BALF of naïve mice treated with SSc-IgG, where neutrophil counts correlated with KC plasma levels. In addition to signs of an inflammatory fibrosis, repeated passive transfer of anti-AT_1_R and anti-ET_A_R Ab-positive SSc-IgG resulted in marked structural alterations of lungs with increased cellular density in interstitial tissue. Our data suggest, furthermore, that these Abs can also activate angiotensin and endothelin receptors across both species because of high receptor homology [34,35].

Our study has some limitations, of which the most prominent is the use of total purified IgG instead of specifically purified anti-AT_1_R and anti-ET_A_R Abs. Instead, we here used receptor antagonists to demonstrate receptor-mediated activation, as previously reported [21,29]. Therefore, measured effects could partly result from other Abs, suggested by incomplete effect inhibition by receptor antagonists. Also, we cannot exclude the participation of other Abs present in IgG on the measured effects. However, we have focused on effects that have already been associated with angiotensin and endothelin-receptor activation. Another shortcoming is that the observed effects showed sometimes very high variability within tested samples, indicating the very complex nature of these Abs.

## Conclusions

In summary, our *in vitro* results indicate an induction of proinflammatory and profibrotic events by anti-AT_1_R and anti-ET_A_R Ab-positive SSc-IgG that might also be present *in vivo*.

Our experimental data complement the association of anti-AT_1_R and anti-ET_A_R Ab to clinical features of SSc, especially with interstitial lung disease. On the basis of these findings, we conclude that anti-AT_1_R and anti-ET_A_R Abs can activate angiotensin and endothelin receptor-expressing cells, among them some of the key players of SSc pathogenesis, and thus affect mechanisms of inflammation and fibrosis. Therefore, anti-AT_1_R and anti-ET_A_R Abs may present a novel future target in SSc therapeutic intervention.

## Abbreviations

Ab: Autoantibody; Abs: autoantibodies; AT1R: angiotensin II type 1 receptor; ATR-A: angiotensin-receptor antagonism; BALF: bronchoalveolar lavage fluid; ECM: extracellular matrix; ETAR: endothelin-1 type A receptor; ETR-A: endothelin receptor antagonism; HMEC-1: human microvascular endothelial cell; IL-8: interleukin-8; NC-IgG: IgG from healthy donors; ROS: reactive oxygen species; SSc: systemic sclerosis; SSc-IgG: Anti-AT_1_R, and anti-ET_A_R, Ab-positive IgG of SSc patients.

## Competing interests

Study was supported by an unrestricted grant from Actelion Pharmaceuticals Germany GmbH; no other competing interests are involved.

## Authors’ contributions

AK, CT, MOB, JG, MR, DD, and GR participated in study design and data interpretation. AK participated in *in vitro* experiments. AK, CT, and RU participated in animal experiments. AK and CT performed statistical analyses. HH provided measurements of anti-AT_1_R and anti-ET_A_R Ab. MWo provided healthy donor-skin samples. MWi provided equipment for BALF analysis. AKü performed histologic preparation of lungs. G-RB, DD, and GR participated in study coordination. AK participated in manuscript preparation with support of all other authors, who read and approved of the manuscript.
